# 7T ^19^F/ ^1^H MRI with perfluorocarbon‐labeled immune cells in pigs: Pilot results with a dedicated twin‐array system with pTX support

**DOI:** 10.1002/mrm.70105

**Published:** 2025-10-10

**Authors:** Maxim Terekhov, Ibrahim A. Elabyad, Marwan A. Hamid, Farzad Jabbarigargari, Rebekka Grampp, Mohammadreza Keshtkar, Anja Stadtmüller, Gabriele Ulm, Sofia Dembski, Anna Frey, Ulrich Hofmann, Wolfgang R. Bauer, Laura M. Schreiber

**Affiliations:** ^1^ Department of Cardiovascular Imaging, Chair of Molecular and Cellular Imaging, Comprehensive Heart Failure Center University Hospital Würzburg Würzburg Germany; ^2^ Chair of Molecular and Cellular Imaging Julius Maximillians‐University Würzburg Germany; ^3^ Department of Internal Medicine I/Cardiology University Hospital Würzburg Würzburg Germany; ^4^ Translational Center for Regenerative Therapies Fraunhofer Institute of Silicate Research (ISC) Würzburg Germany

**Keywords:** ^19^F MRI, inflamation, parallel transmit, ultra‐high‐field

## Abstract

**Purpose:**

Cardiac inflammation plays a key role in many diseases. However, its underlying mechanisms and progression remain poorly understood. Using an ultra‐high field (UHF) may increase the sensitivity of MRI of inflammatory processes with ^19^F‐labeled immune cells. The high Larmor frequency of ^19^F (∼95% of that for ^1^H‐nuclei) leads to similar technical hurdles in acquiring MR images at UHF that originate from the heterogeneity of B_1_^+^. We aimed to develop a system of transmit/receive (Tx/Rx) arrays exploiting parallel transmit (pTX) technology for B_1_^+^‐shimming to acquire a combination of high‐quality anatomical ^1^H MR‐images of a pig heart and ^19^F images of labeled immune cells at 7T.

**Method:**

The 16‐element twin‐arrays for ^1^H and ^19^F nuclei were designed using electromagnetic simulations with a focus on optimal B_1_‐shimming and g‐factor in the region of the pig heart. pTX support allows for the subject‐specific B_1_‐shimming for ^1^H cardiac MRI and transfer of settings for the B_1_‐shimming to the ^19^F twin‐array.

**Results:**

The twin‐array system was implemented and tested in a pig‐thorax‐‐shaped phantom, in‐vivo in a myocardial infarction pig model, and in an excised heart. A transfer of static B_1_+ shimming setting between the ^1^H to ^19^F arrays was demonstrated. The parallel imaging acceleration of up to a factor 4 was possible with a g‐factor <1.3 for both arrays. The 7T MRI of ^19^F‐labeled immune cells in the heart of the pig was demonstrated both in‐vivo and ex‐vivo.

**Conclusion:**

The 7T MRI of perfluorocarbon‐labeled immune cells in a large‐animal myocardial infarction model becomes feasible using a novel dedicated twin‐arrays system.

## 1 INTRODUCTION

Cardiac inflammation plays a key role in many cardiovascular diseases, yet its underlying mechanisms and progression remain poorly understood. Cardiac MRI with immune cells (ICs) labeled by nanoparticles incorporating ^19^F atoms offers a promising approach to directly visualize and monitor inflammatory responses in the heart.^1^, ^2^, ^3^, ^4^, ^5^ Employing ultra‐high‐field (UHF) cardiac MRI has the potential to significantly increase the SNR of MRI, and in the past decade was successfully used for the cardiac imaging of both humans^6^ and large animals.^7^, ^8^, ^9^ In the context of IC‐tracking, 7T MRI may enable a significant increase in the spatial resolution of both ^19^F‐MRI and underlying anatomical ^1^H images. It also promises better localization of ICs compared to imaging at lower field strengths. The close Larmor frequency of ^19^F and ^1^H nuclei leads to similar technical hurdles for ^19^F MRI in acquiring cardiac images at UHF that originate from the heterogeneity of B_1_^+^ distribution and the necessity of using dedicated Tx/Rx arrays exploiting parallel transmit (pTX) technology.^10^, ^11^, ^12^, ^13^, ^14^ The pig thorax shape differs quite significantly from that of humans. Thus, a dedicated design of the RF arrays appears to be needed to enable efficient B_1_^+^‐shimming and optimal SNR in the heart.^11^, ^15^

In this work, we describe the simulation, design, implementation, and test of a dedicated RF‐arrays system for cardiac MRI images in the pig model of myocardial infarction (MI) for both ^19^F and ^1^H nuclei in a whole body 7T MR‐scanner with pTX support. We test the arrays in ^19^F‐MRI of perfluorocarbon‐labeled ICs in vivo in a MI pig model, and ex‐vivo using a setup for imaging of the explanted hearts.

## 2 METHODS

**FIGURE 1:**
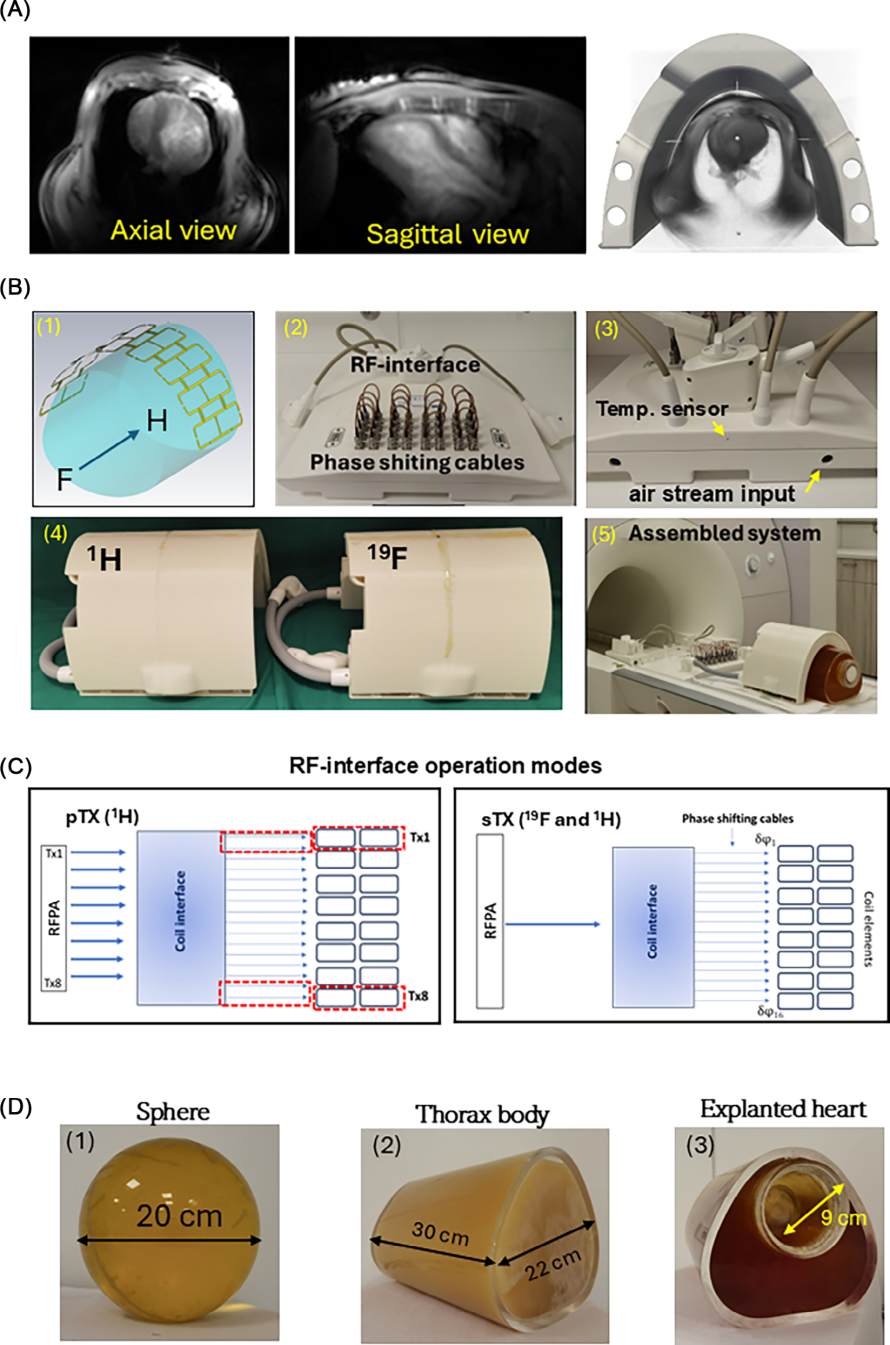
(A) Design of the array's shape based on the MRI images of a pig thorax of the targeted weight range (20–40 kg). The key features of in‐house system development included (1) array elements design (identical for ^1^H and ^19^F arrays) (2) the concept of dual‐mode RF interface with additional cooling and temperature control at long time/low duty cycle operation and (3) dedicated shape housings for both arrays (C) With the customized RF interface the RFPA B_1_^+^ shimming for ^1^H‐array can be performed in pTX operation mode. To form 8Tx/16Rx configuration in pTX mode, the 16 elements are connected to RFPA pairwise. In sTx operation mode for each element, the individual phase‐shifting cable could be connected, enabling a phase‐only B_1_^+^‐shimming vector for both ^1^H and ^19^F arrays. (D) Testing and adjustment of the array were done using spherical (D.1) and thorax phantoms (D.2, D.3) from acrylic glass. For the explanted heart measurements, the thorax phantom with inner bore was used (D.3).

The design of the arrays' concept, electromagnetic (EM) simulations, printed circuit board (PCB), and coil housing were done in‐house. The EM simulations were done using CST Microwave Studio (v 2019–2021, Dassault, France) with subsequent postprocessing using in‐house developed Matlab (Mathworks, USA) and Python scripts. The 16‐element PCB of the array is allocated on the housing, fitting the shape of the pig thorax (Figure 1A). The housing was designed based on the shape of mono‐surface arrays, which demonstrated efficient cardiac MRI of pigs.^11^ The specific dimensions of the housing were selected for pigs of 25‐40 kg. The tight fitting of the housing to the pig thorax shape allows for a sufficiently precise reproduction of the position of the arrays relative to the animal anatomy when ^1^H and ^19^F arrays are exchanged during the measurements.

The array elements are designed as eight pairs of rectangular loops connected by a shared conductor, as shown in Figure 1B panel 1. The total length of the loop pair in head‐‐foot direction is 25 cm, and the width is 5.5 cm. For the EM‐simulation, the array's models were loaded with the model of phantoms also implemented physically, for the subsequent validation and testing of the array by MRI experiments (Figure 1D).

With the customized RF interface, the ^1^H‐array can be used in both 8Tx/16Rx pTX‐mode and 1Tx/16Rx in single Tx (sTx) “combined” mode. To form an 8Tx configuration in pTX mode, the 16 elements are connected pairwise (Figure 1B,C). In pTX mode, each loop of the pair is driven by the voltage of the respective RFPA channel (equal phase and magnitude for both loops). In combined sTx mode, all 16 elements are connected to the power splitter of the RF interface. Because pTX‐support for X‐nuclei is not implemented by vendors of 7T MR‐scanners, the ^19^F‐array application is limited to sTX‐mode, and B_1_^+^‐shimming is enabled by phase‐shifting cables connected to the respective sockets for each of 16 array elements (Figure 1B, panel 2). The general schematics of the connection coil and RF interface, and using RFPA and phase‐shifting cables for B_1_^+^ shimming in pTX and combined modes, respectively, are depicted in Figure 1C.

The implementation of the RF interface and tuning/match circuitry was done by RAPID Biomedical (Rimpar, Germany). The average RF power passing the power splitter by using sequences with a high duty cycle (e.g., balanced SSFP [bSSFP]^16^) may exceed 100 W. This required the implementation of an external cooling of RF components by the stream of pressurized air supplied through the dedicated openings in the interface housing. Additionally, the opening for inserting a fiber‐optical sensor was introduced to control the temperature of the power combiner heavily loaded in the sTX operation mode.

The design of the interface in combination with identical shapes of ^1^H and ^19^F arrays allows for implementing a “twin‐arrays” concept in the context of B_1_^+^ shimming. The acquisition of B_1_^+^ maps of individual transmit channels for subject‐specific B_1_^+^shimming is only technically possible by operation in pTX mode and, thus, supports only ^1^H nuclei. However, because of the close Larmor frequencies of ^1^H and ^19^F and the identical design of the two arrays, the B_1_^+^ shimming vector computed for the ^1^H array can also be applied to the ^19^F array, providing identical or very close results. In this work, the acquired relative B_1_^+^ maps of eight Tx‐channels for ^1^H array in combination with a “phase‐only” regime for B_1_^+^ optimization allowed for computing an optimal “phase‐only” transmit vector with eight elements. These vectors were implemented in the form of 16 phase‐shifting cables equal for each two elements of the paired loop, which were connected to the respective sockets on the interface (Figure 1B). The cables of necessary length could be assembled within several minutes using the collection of cables with predefined phase shift stripped together by the BNC connectors.

In this way, the B_1_^+^ shimming was applied in sTx mode for both ^1^H and ^19^F arrays. The design of arrays and RF interface (individual phase shifting cables for each of 16 loops) allows for additional degrees of freedom for the B_1_^+^ shimming in the sTX mode using phase vectors with 16 different components.

The perfluoro‐cron‐ether (PFCE) nano‐emulsion (NE) was produced in‐house (ISC, Würzburg). The particle shell consisted of the lipid shell^1^ covering the core, which encapsulates 20% of the volume of PFCE. The average size of the particles measured by ultrasound scattering was ≈ 260 nanometers.

The homogeneous phantoms used for validation of EM‐simulation and initial testing of arrays were (i) acryl sphere with 200 mm diameter (Figure 1D.1) and (ii) custom‐designed phantom of pig thorax reproducing the inner shape of the coil housing (Figure 1D.2). The second dedicated phantom for measuring explanted hearts (Figure 1D.3) has an inner bore that allows positioning container with the heart placed in the fixative formalin or the NaCl solution. The composition filling the phantoms includes polyvinylpyrrolidone (PVP) solution prepared as described in^11^ and mimicking the electromagnetic properties of muscles. To enable ^19^F imaging, the PFCE‐NE was added to the composition of the spherical and pig thorax phantoms, providing an emulsion concentration ≈1:10. This enabled the concentration of ^19^F atoms sufficient to acquire gradient echo (GRE) images with SNR ≈10 within 5–7 min. To reconstruct reliable B_1_^+^ maps with ^19^F signal, a higher SNR was required. To ensure it, the cylindrical container of 80 mm diameter with 1:1 mixture of PVP and PFCE‐NE was placed in the inner bore of the body phantom (Figure 3A). Moreover, the B_1_^+^‐ shimming applied to the region surrounded by air cavity imitates partially the in‐vivo situation of the heart surrounded by lungs having significantly lower tissue density and electrical permittivity.

All MRI measurements were done using a 7T Magnetom™ “Terra” scanner (Siemens Healthineers, Germany). The B_0_ shimming was performed using ^1^H signal and copied to the shim system for ^19^F scans using the vendor‐integrated utility. The B_1_^+^ mapping measurements in spherical phantom for the comparison with EM‐simulations were performed using a radial‐phase encoded gradient echo sequence with channel‐wise excitation as described in,^17^, ^18^ and a saturated turbo‐FLASH pulse sequence with interferometry reconstruction.^19^ The absolute B_1_‐mapping in the body phantom with the inner bore was performed using a pre‐saturated turbo‐FLASH‐based “B_1_‐sandwich” sequence.^20^ The coil characterization for the noise amplification factor of parallel imaging (“g‐factor” maps) and SNR was performed with the vendor's “coil‐utility” sequence. The “g‐factor” maps were generated by the vendor‐integrated routine. In‐vivo cardiac MRI for ^1^H was done using a vendor‐provided GRE CINE pulse sequence (BEAT). An acoustic system (ACT, MRI Tools, Germany) was used for cardiac gating. ^19^F MRI was performed using GRE sequence. The explanted hearts were placed in a water‐NaCl solution to avoid susceptibility artifacts. To suppress the surrounding water and to provide optimal contrast of the scar tissue, a double‐inversion recovery (DIR) GRE sequence (MP2RAGE) was used for ^1^H imaging. The key sequence parameters for all sequences are summarized in Table S1 of the Supporting Materials.

The static B_1_^+^ optimization was performed using an in‐house developed B_1_^+^‐shimming toolbox. Cost functions optimizing B_1_^+^‐efficiency and homogeneity (individually and in combination) were employed as described in previous works on the design of a dedicated coil for cardiac MRI in humans and pigs at 7T.^15^, ^21^, ^22^, ^23^, ^24^ The acquired relative B_1_+ maps of individual Tx‐channels were reconstructed as described in.^17^ The global Differential Evolution solver (scipy. optimize v.1.10 package) was used for searching optimal transmit vectors. The average computation time for 16‐component pTX‐mode vectors (magnitude and phase of the RFPA voltage) optimizing B_1_+ in the region of interest defined as a cylindrical volume tightly enclosing an average‐sized pig heart was ≈80 s. For the eight‐component “phase‐only” Tx‐vector, the computation time was ≈60 s. The setting of the pTX Tx‐vector to the RFPA in pTX mode was done using the pTX adjustment platform of the scanner.

Pilot in vivo scans were performed using a MI model of the pig produced with permission of the local Animal Care Committee (RUF‐1933) as described in.^8^, ^9^ The induction of myocardial injury was performed by 90‐min occlusion of the left anterior descending coronary artery using a balloon catheter, and subsequent reperfusion. The 75 mL of PFCE‐NE was injected 2 days after the induction of MI, and measurement was performed at day 5 after the injection. The total duration of ^1^H MRI (B_1_^+^‐shimming, CINE with multiple shimming settings, late gadolinium enhancement [LGE]) was ≈1.5 h. The individual ^19^F images using GRE sequence were measured from ≈10 (thorax overview) to ≈20 min (dedicated heart). The transmitter reference voltage for in‐vivo ^19^F scans was set based on the measurements in the thorax phantom.

The retrieval of the heart with its subsequent MR measurements was done ≈1 h after the euthanasia on day 15 after MI. The heart was scanned fresh and placed in NaCl solution and later was fixed in a 4% formalin solution for the high‐resolution scan tests.

All data post‐processing was performed using in‐house developed Matlab scripts. For simplifying the registration, the positioning of slices for ^19^F acquisition was done using the previously acquired ^1^H images and setting the same centers of slices and dimensions of FOV. The overlaying of the ^1^H and ^19^F images was done by adjusting the transparency of the output of both images in the same graphical context.

## 3 RESULTS

The twin‐array concept was proven by EM simulations. Optimized choice of tuning/matching capacities provides optimal RF design of each array, whereas identical geometry of both arrays allows for transferring the phase shift vector providing the B_1_^+^‐shimming from the ^1^H to the ^19^F‐array.

**FIGURE 2:**
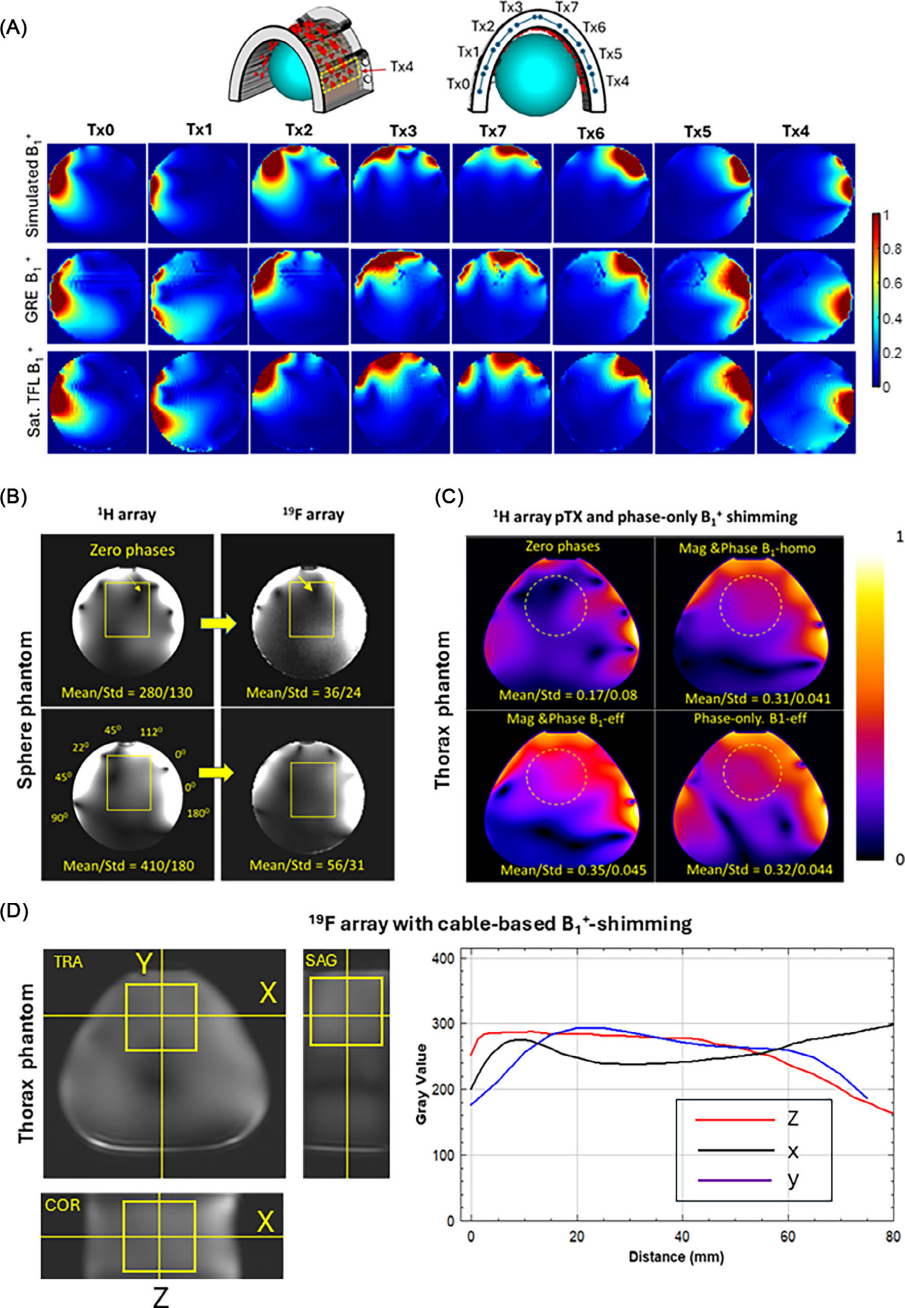
(A) Validation of EM‐simulated B_1_^+^ maps in the spherical phantom. Simulated and experimental normalized relative B_1_^+^ maps demonstrate good qualitative agreement for most of the individual Tx‐channels. (B) Example of phase‐only B_1_^+^‐shimming transfer between ^1^H and ^19^F arrays in the spherical phantom. The phase vector used for both shimming is shown in the ^1^H image. Arrows label the destructive B_1_^+^‐interference sites that are efficiently removed by B_1_^+^‐shimming (C) The B_1_^+^‐shimming using full‐pTX (magnitude & phase) and phase‐only approaches in the pig thorax phantom. (D) Transfer of B_1_^+^+ shimming to ^19^F array. The flatness of the RF‐excitation profile (via GRE signal intensity profile) is shown on the right panel.

Figure 2A shows validation of the EM simulation of the ^1^H array using a spherical phantom and channel‐wise B_1_^+^ mapping with two different pulse sequences. We observed good agreement between simulated and measured B_1_^+^ maps of individual Tx channels. The localization of the maxima of magnitudes of the B_1_^+^ profiles corresponds well to the position of the paired element rows around the phantom. The maxima of intensity of the B_1_^+^ maps are sufficiently distinct in the center of the phantom. Thus, an efficient shaping of targeted combined B_1_^+^ distribution using pTX technique appears feasible.

Figure 2C demonstrates the capability of transferring B_1_^+^ shimming between twin arrays in the spherical phantom. Figure 2D demonstrates the effect of subject‐specific B_1_^+^‐shimming in the pig body phantom in a region roughly corresponding to the heart position. As expected, using full pTX capabilities with manipulation of phases and magnitudes of the transmitter voltages allows for essential improvement of B_1_^+^ efficiency and homogeneity. The values of mean B_1_^+^ and SD before and after applying B_1_^+^‐shimming are drawn in the figure. However, the phase‐only B_1_^+^‐shimming regime using cables also demonstrates a sufficient effect of B_1_^+^ optimization in the targeted region, with similar improvement factors for both mean B_1_^+^ and its coefficient of variation.

**FIGURE 3:**
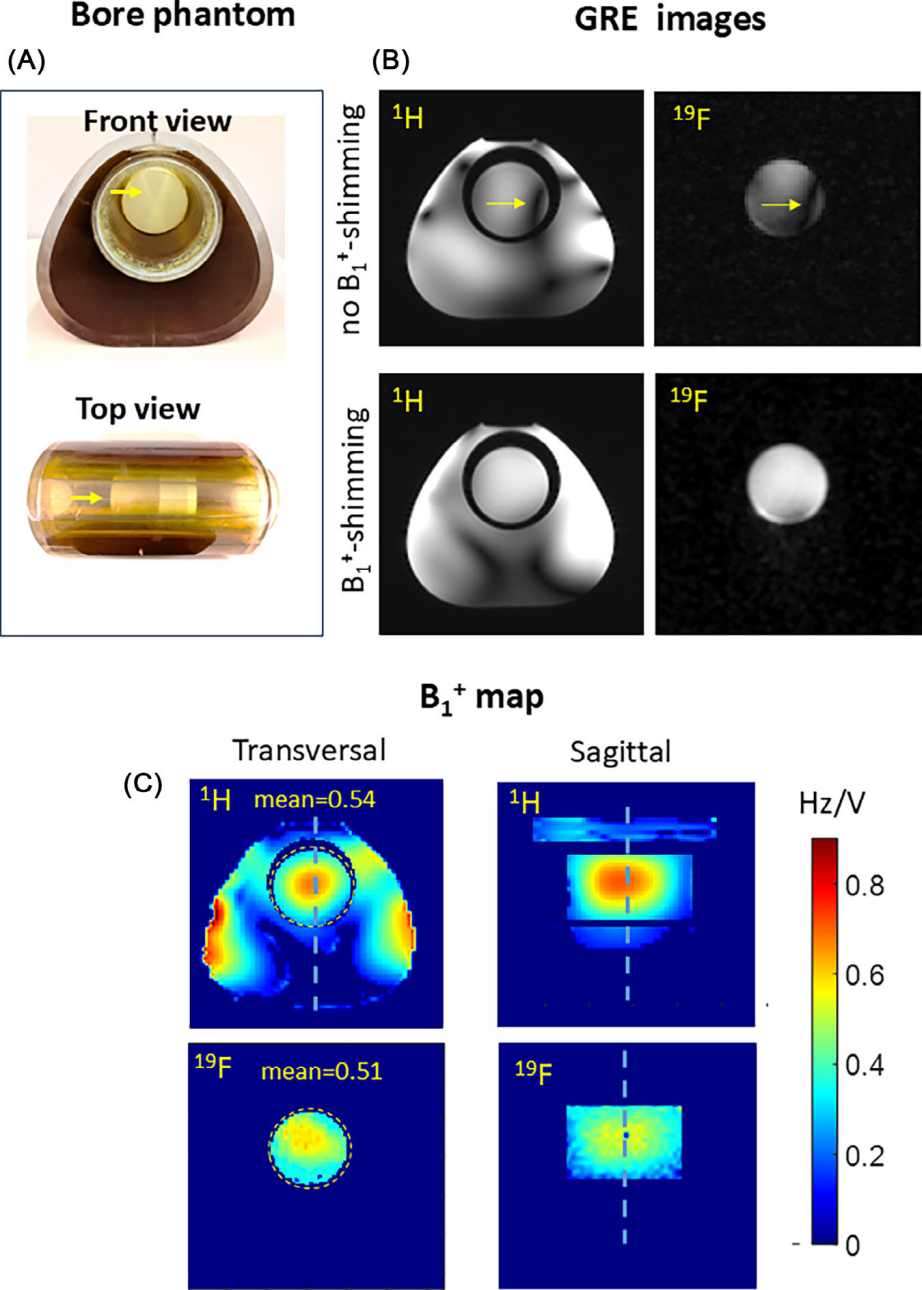
(A) The positioning of the ^19^F phantom in the bore of the pig thorax phantom. The cylinder with a high concentration of PFCE‐NE (labeled by arrows) enables sufficient SNR for the absolute ^19^F B_1_^+^ mapping after B_1_^+^‐shimming in the region corresponding to the pig heart. (B) The 1H and 19F GRE images before and after connecting phase cables, enabling B_1_^+^ shimming targeted for B1+ efficiency. The elimination of the B_1_^+^ artifact and the increase of the signal with applied shimming are well seen. (C) The absolute B_1_^+^ maps of ^1^H and ^19^F arrays with the same B_1_^+^‐shimming applied (slice positions are labeled by dashed lines). Despite the presence of multiple interfaces between substances with different electromagnetic properties (air, PVP, and PVP mixed with PFCE‐NE emulsion) B_1_^+^ maps show a good agreement in both transversal and sagittal views.

Figure 3 shows the capability to perform transfer of the B_1_^+^ shimming settings from ^1^H to ^19^F array performed for the cylindrical volume in the air cavity (Figure 3A). The GRE images without B_1_^+^‐shimming show distinct destructive interference artifacts in the container with PVP‐NE composition placed inside the bore, observed almost identically in ^1^H and ^19^F images. This artifact is efficiently eliminated by phase‐only shimming applied for ^1^H array (left side panels). The same phase‐only settings enable efficient removal of destructive interference for the ^19^F array as demonstrated by both GRE images and the absolute B_1_^+^ map (Figure 3C). Overall, a good match is observed between both B_1_^+^‐maps in both transversal and sagittal planes, despite probably not having a complete electromagnetic equivalence of ^1^H and ^19^F array for the complex geometries of the loading with the inner bore.

**FIGURE 4:**
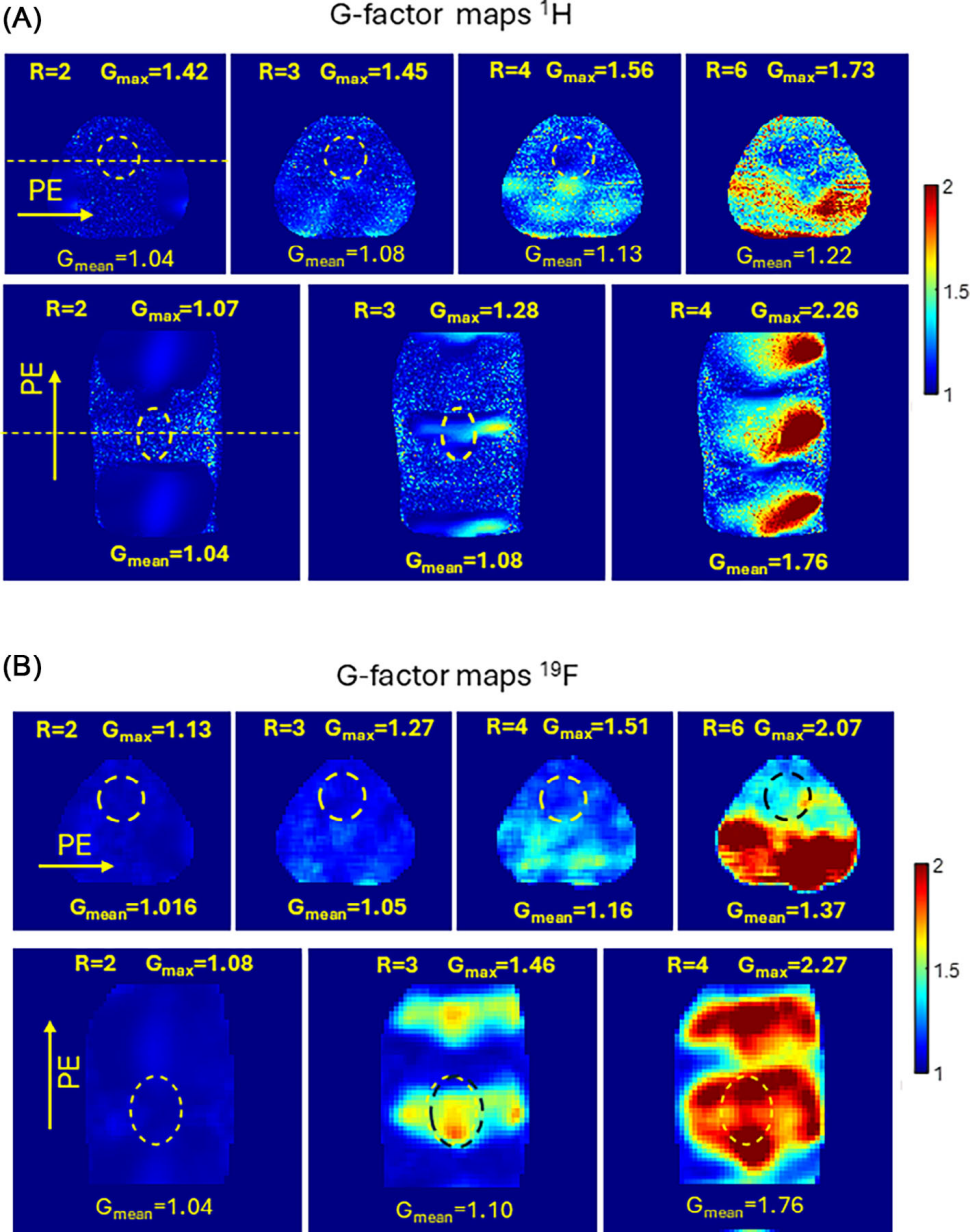
Measured g‐factor maps of ^1^H (A) and ^19^F (B) arrays with phase encoding in LR and HF directions. The statistical metrics show that the left‐right phase‐encoding direction most often used in cardiac MRI is the acceleration factor up to R=4 with a very moderate g‐factor penalty (G_mean_=1.16). The same is true for the ^19^F twin‐array where acceleration can be efficiently used for keeping scan time in the range of the single breath‐hold, minimizing motion artifacts.

Figure 4 shows a measured g‐factor in the pig body phantom. The B_1_^+^ shimming for both arrays was applied before the data acquisition. Figure 4A demonstrates that at ^1^H parallel imaging acceleration up to factor 4 in the left–right direction and up to factor ≈3 in the head‐‐feet direction appears feasible at a moderate mean and maximal g‐factor (g_mean_ <1.25 and g_max_ <2). In particular, the maximal value of the g‐factor in the heart region for the left‐to‐right PE direction does not exceed 1.56, while the mean value is 1.13. Figure 4B confirms similar results for ^19^F array.

**FIGURE 5:**
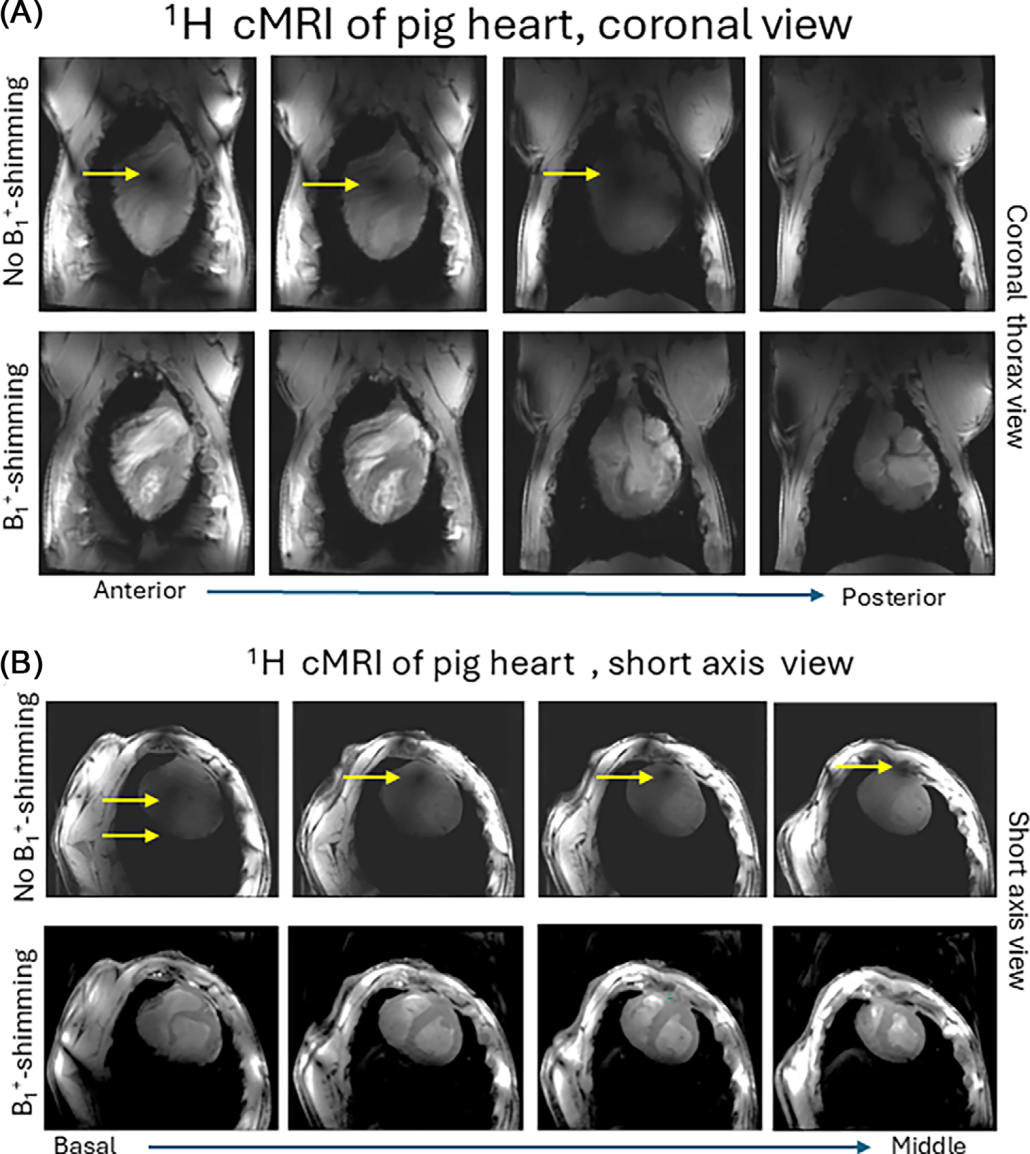
Cardiac MRI using ^1^H‐array. The efficiency of the subject‐specific B_1_+‐shimming is confirmed by the elimination of the destructive interferences and a significant increase of Tx‐efficiency on both coronal planes (A) and short‐axis view images (B). The suppression of B_1_^+^‐artifacts (arrows) and strong gain of B_1_^+^ penetration at the posterior wall is observed.

Figure 5 demonstrates the capability of the ^1^H array for the in vivo ^1^H cardiac MRI, both in terms of B_1_^+^ shimming and parallel imaging. Figure 5A,B show images with coronal and short‐axis views, respectively, acquired with zero phases and equal magnitudes of Tx‐channels (“no B_1_^+^‐shimming”) and with B_1_^+^ shimming applied. Additionally, supplementary material Video S1 demonstrates long‐axis view movies of CINE measured with geometry‐based and different subject‐specific B_1_^+^ shimming. The supplementary Video S2 and Figure S1 show CINE movies of the selected slices of the short‐axis heart view and LGE images with B_1_^+^ shimming applied.

**FIGURE 6:**
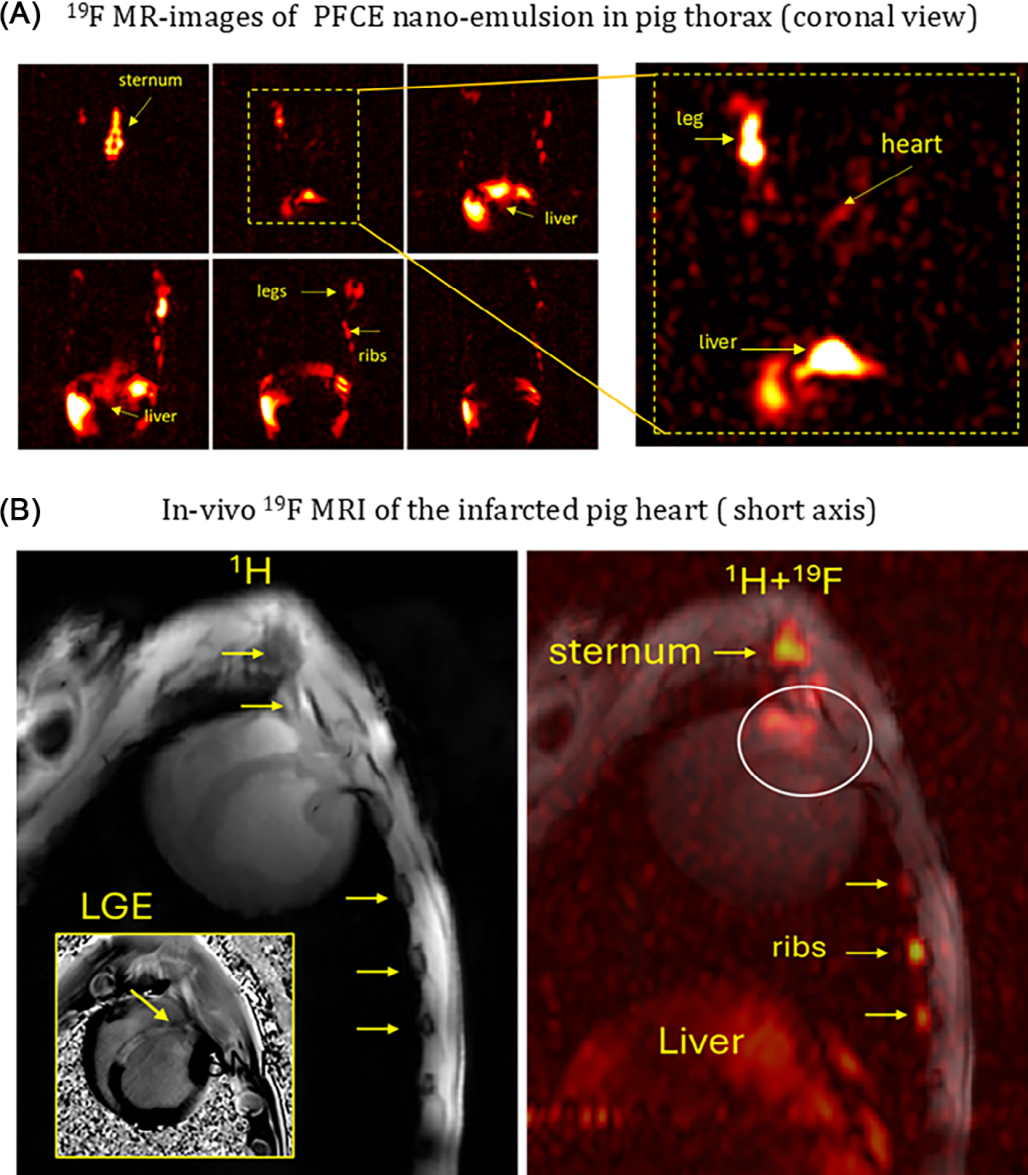
(A) Overview ^19^F GRE MR‐images (“hot” colormap) of PFCE‐NE in the pig thorax. Acquisition time ∼20 min with pixel resolution 5 × 5 × 8 mm (200 mm^3^). The highest ^19^F NE concentrations are detected in the liver and bone marrow, which are known to be large deposits of ICs in the body. (B) The in‐vivo ^1^H (left panel) and ^19^F (right panel) MRI of cardiac inflammation after reperfusion injury. The infarcted area in the septum and the part of the left and right ventricular myocardium near its junction could be seen on LGE image (left panel). The ^19^F signal is seen in the heart at the junction of the left and right ventricular walls (labeled by the white circle). This signal presumably originates from the PFCE‐NE consumed by the ICs concentrated in the inflammation foci in the infarcted area.

Figure 6 depicts the pilot 7T ^19^F MR‐images of PFCE‐labeled NE in the pig body measured in‐vivo. Figure 6A shows an overview ^19^F MR‐image of the whole thorax. The highest concentration of ^19^F NE was detected in the sternum, liver, and bone marrow of ribs and extremities (legs). Figure 6B shows the detection of the ^19^F signal in the region of MI at the junction of the left and right ventricular walls. This signal presumably originates from the ICs that are localized at the time of imaging in that region.

**FIGURE 7:**
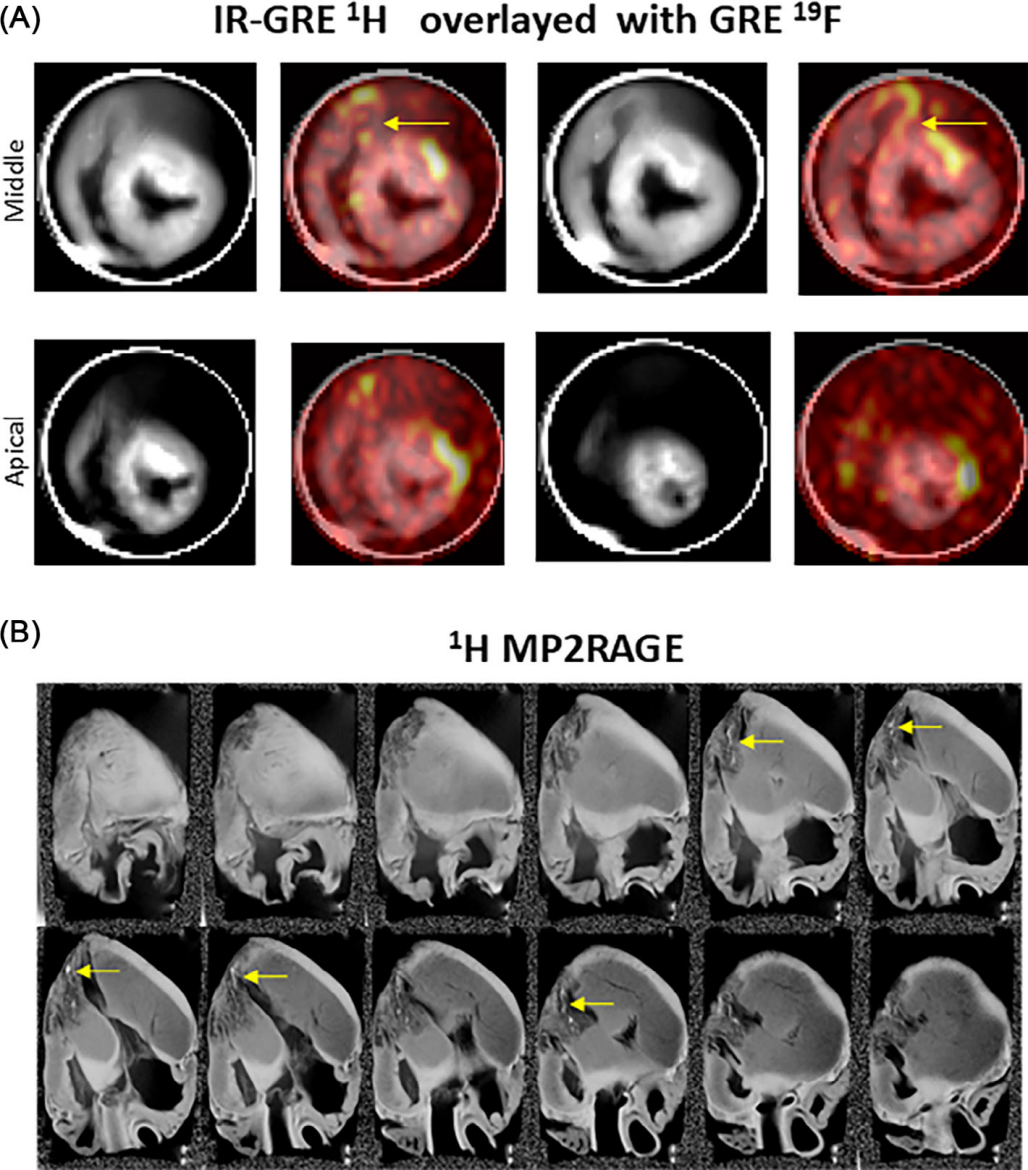
(A) The anatomical ^1^H IR‐GRE images (“grayscale” lookup table) and overlayed ^19^F GRE images (“hot” lookup table) of the fresh non‐fixed explanted heart placed in the water bath were acquired with 5 × 5 × 8 mm (200mm^3^) voxel size. The ^19^F signal of the PFCE NE consumed by ICs is observed at the junction of the left and right ventricle wall (yellow arrows) that roughly corresponds location observed in the in‐vivo image (Figure 6B). (B) The high‐resolution images (0.65mm isotropic voxel size) of the fixed explanted heart acquired witha ^1^H array. The dedicated B_1_^+^ shimming inside the bore of the loading phantom (Figure 1D.3) allows for double‐IR GRE (MP2RAGE) acquisition and reconstruction. This enables a good contrast for visualizing the scar tissue and remaining Gd‐contrast in ^1^H images (yellow arrows).

Figure 7 demonstrates the MRI of explanted pig hearts. Figure 7A shows images of a fresh excised heart scanned within the first 60 min after the retrieval to preserve tissue structure and minimize the effects of autolysis of the tissue. B_1_^+^ shimming was performed before in full pTX mode at ^1^H Larmor frequency and subsequently used in phase‐only mode in ^19^F scans. Inversion recovery allowed the suppression of the background water bath signal in ^1^H images. The ^19^F signal of the PFCE‐NE consumed by ICs is localized in the regions of the visible tissue alteration due to the infarction (hypo‐intense on the T_1_‐weighted images). The localization of the ^19^F‐signal in the excised heart matches well with the position observed in vivo (junction of the anterior left and right ventricular wall).

Figure 7B shows high‐resolution 3D MP2RAGE MR‐images of the fixed heart acquired with the ^1^H array after B_1_^+^ shimming. Isotropic spatial resolutions of 0.65 mm for ^1^H with an acquisition time of ≈25 min allow for detailed analysis of the anatomical distribution of the scar formed after the MI and reveal residual LGE contrast in the fibrotic tissue.

## 4 DISCUSSION

We present the successful development and test of a twin‐array system for ^1^H and ^19^F nuclei in pigs. Our concept enables optimal transmission and reception at both nuclei while allowing seamless transfer of B_1_^+^‐shims settings from ^1^H to ^19^F. The custom‐designed housing for the twin ^1^H and ^19^F arrays, shaped to match the realistic pig thorax geometry, ensures a close‐fitting arrangement of loop elements around the heart, thus improving both SNR and capability for B_1_^+^‐shimming in the heart region. The identical geometry of the two arrays addresses a key technical challenge associated with the ^19^F array, which was enabling subject‐specific B_1_^+^‐shimming despite the scanner's limitation of sTx mode for X‐nuclei at 7 T.

The ^1^H array demonstrated a good performance of subject‐specific static B_1_^+^‐shimming performed using pre‐acquired B_1_^+^ maps for ^1^H array. A factor 1.8 increase of the mean signal and a factor 3.6 decrease in the coefficient of variation in the homogeneous pig thorax phantom were achieved compared to the non‐shimmed state. The B_1_^+^ maps acquired in pTX operation mode for ^1^H array were then used to configure the length of cables to obtain optimal B_1_^+^‐shimming for the ^19^F array. Using phantoms applicable for both ^1^H and ^19^F imaging, we demonstrated that the B_1_^+^ shaped by phase‐only static B_1_‐shimming for the ^1^H array could be effectively transferred and reproduced by the ^19^F array.

In the pig body‐mimicking phantoms (with and without the inner bore), the transfer of ^1^H B_1_^+^ shimming to ^19^F array enables eliminating destructive interference artifacts and getting quite homogeneous ^19^F signal excitation in the region corresponding to the pig heart position. A reasonable agreement of absolute B_1_^+^ maps for ^1^H and ^19^F arrays was demonstrated in both transversal and sagittal planes. The discrepancies in the excitation profiles between ^1^H and ^19^F observed in the posterior region of body phantoms most probably originate from an incomplete electromagnetic equivalence of both arrays, which is difficult to achieve technically. However, these discrepancies were not critical for cardiac MRI application because at the location corresponding to the heart region, the B_1_^+^ shimming setting transferred from ^1^H to ^19^F array allows for efficient elimination of the destructive B_1_^+^ interferences and enables a flat and relatively homogeneous excitation profile for ^19^F array. The quality of B_1_^+^‐shimming transfer between ^1^H and ^19^F arrays could be improved further by fine‐tuning all 16 phase shifters to compensate for the discrepancies. Potentially, this fine‐tuning could be done using B_1_^+^‐maps of each of 16 elements acquired individually by connecting them one by one using the RF‐interface sockets. This approach, however, is not practical for the in vivo application due to the logistical efforts needed to switch all cables one by one and the relatively long time needed to acquire 16 B_1_^+^‐maps. It can be made feasible, for example, using pig cadavers to optimize the phase shifts within elements in the pair or design a universal 16‐component Tx‐vector as described in previous works.^15^, ^21^, ^23^

The static B_1_^+^‐shimming capability of the ^1^H array was successfully demonstrated in the in‐vivo cardiac MRI by efficient removal of destructive interferences observed when using “zero shimming” or “geometry‐based” shims and enabling a good blood‐to‐tissue contrast of CINE‐imaging. Notably, the LGE images (Figure S1) clearly show enhancement in areas of post‐MI scar tissue in the posterior walls of apical slices. The high quality of LGE visualization proves an efficient magnetization inversion over the heart, including a posterior cardiac wall, enabled by the strong B_1_^+^‐field homogeneity and transmission efficiency of the surface Tx/Rx arrays at 7T. The array's design with 16 Rx elements allocated around a pig thorax allows for efficient use of parallel acquisition and reconstruction for the heart region. This enables a good quality of in vivo ^1^H CINE cardiac MRI and other cardiac imaging modalities.

The designed twin‐array system proved its effectiveness for high‐resolution imaging of explanted hearts using a specialized phantom with an inner bore. This phantom provides sufficient electrical loading of the arrays and precise positioning of the heart container for B_1_^+^‐shimming, ensuring sufficient RF power delivery for inversion pulses. This setup, in combination with a thoroughly done subject‐specific B_1_^+^ shimming, allowed for the efficient use of inversion recovery for suppressing a background signal in the samples placed in a water environment to minimize susceptibility artifacts. Furthermore, it enables visualization of residual contrast agents remaining in scar tissue regions after LGE experiments.

## 5 CONCLUSIONS

In this work, we demonstrated the design and initial testing of the twin‐array system, which allowed us to perform the first ^19^F/^1^H MRI experiments in the pig heart at 7T, using a whole‐body human medical MR‐scanner. This design enhances the efficiency and homogeneity of ^19^F excitation, addressing a key challenge in ultra‐high‐field imaging. The ^19^F/^1^H array system demonstrated a strong potential for highly sensitive detection of ^19^F‐labeled cells in the heart and other organs while simultaneously providing high‐resolution ^1^H images. This capability ensures precise co‐localization of labeled NE distributions with the underlying anatomical structures, making it a valuable tool for advancing cardiac imaging and IC tracking applications.

## Supporting information


**Video S1.** Demonstration of B^1^_+_ shimming effect for ^1^H‐array on the long‐axis CINE images. Panel (1) – geometry‐based phase‐only shimming shows a strong B_1_^+^‐artefact. Panel (2) – shimming targeted on B_1_^+^ efficiency. Panels (3) – shimming targeted for B_1_^+^ homogeneity. Panel (4) – balanced shimming targeted on both B_1_^+^ homogeneity and efficiency as described in (23).

**Video S2.** Individual slices of the short‐axis 3D stack acquired with the CINE pulse sequence. 30 cardiac phases are reconstructed retrospectively. The positions of the slices are shown on the long axis view (top panel). The applied B_1_^+^ shimming optimizes homogeneity (see Figure S1) and provides a relatively homogeneous blood‐to‐tissue contrast throughout the volume of the heart.

**Figure S1** The Late‐Gadolinium Enhancement MR‐images of a pig model of acute myocardial infarction. B_1_^+^ shimming targeted to maximize B_1_^+^‐efficiency was applied. Images show well‐visible enhancement in the area of post‐MI scar spreading in the septum in mid‐myocardial slices and enters the anterior and posterior walls in the apical slices (yellow labels). The latter confirms sufficient efficiency of magnetization inversion, which is usually problematic to ensure using surface cardiac Tx/Rx arrays at 7T.

**Table S1.** Pulse sequences and their parameters used for the experimental MR‐data acquisition.

## References

[1] Flogel U , Ding Z , Hardung H , et al. In vivo monitoring of inflammation after cardiac and cerebral ischemia by fluorine magnetic resonance imaging. Circulation. 2008;118:140‐148. 18574049 10.1161/CIRCULATIONAHA.107.737890 PMC2735653

[2] Flogel U , Temme S , Jacoby C , et al. Multi‐targeted (1)H/(19)F MRI unmasks specific danger patterns for emerging cardiovascular disorders. Nat Commun. 2021;12:5847. 34615876 10.1038/s41467-021-26146-6 PMC8494909

[3] Rothe M , Jahn A , Weiss K , et al. In vivo (19)F MR inflammation imaging after myocardial infarction in a large animal model at 3 T. MAGMA. 2019;32:5‐13. 30421248 10.1007/s10334-018-0714-8

[4] Bonner F , Gastl M , Nienhaus F , et al. Regional analysis of inflammation and contractile function in reperfused acute myocardial infarction by in vivo (19)F cardiovascular magnetic resonance in pigs. Basic Res Cardiol. 2022;117:21. 35389088 10.1007/s00395-022-00928-5 PMC8989832

[5] Ye YX , Basse‐Lusebrink TC , Arias‐Loza PA , et al. Monitoring of monocyte recruitment in reperfused myocardial infarction with intramyocardial hemorrhage and microvascular obstruction by combined fluorine 19 and proton cardiac magnetic resonance imaging. Circulation. 2013;128:1878‐1888. 24025595 10.1161/CIRCULATIONAHA.113.000731

[6] Reiter T , Lohr D , Hock M , et al. On the way to routine cardiac MRI at 7 tesla – a pilot study on consecutive 84 examinations. PLoS One. 2021;16:e0252797. 34297720 10.1371/journal.pone.0252797 PMC8301632

[7] Terekhov MLD , Hock M , Bille M , et al. Visualization of post‐infarction cardiac tissue remodeling at 7T using $T_{2}^{*}$ methodology: longitudinal pilot study in a porcine myocardial infarction model. Proceedings of the International Society for Magnetic Resonance in Medicine Annual Meeting; International Society of Magnetic Resonance in Medicine; 2021.

[8] Schreiber LM , Lohr D , Baltes S , et al. Ultra‐high field cardiac MRI in large animals and humans for translational cardiovascular research. Front Cardiovasc Med. 2023;10:1068390. 37255709 10.3389/fcvm.2023.1068390 PMC10225557

[9] Lohr D , Kollmann A , Bille M , et al. Precision imaging of cardiac function and scar size in acute and chronic porcine myocardial infarction using ultrahigh‐field MRI. Commun Med (Lond). 2024;4:146. 39026075 10.1038/s43856-024-00559-y PMC11258271

[10] Elabyad IA , Terekhov M , Bille M , Schreiber LM . Design and implementation of two 16‐element antisymmetric transceiver coil arrays for parallel transmission human cardiac MRI at 7 T. IEEE Trans Microw Theory Techn. 2021;69:3540‐3557.

[11] Elabyad IA , Terekhov M , Lohr D , Stefanescu MR , Baltes S , Schreiber LM . A novel mono‐surface antisymmetric 8Tx/16Rx coil Array for parallel transmit cardiac MRI in pigs at 7T. Sci Rep. 2020;10:3117. 32080274 10.1038/s41598-020-59949-6 PMC7033245

[12] Elabyad IA , Terekhov M , Stefanescu MR , Lohr D , Fischer M , Schreiber LM . Design of a novel antisymmetric coil array for parallel transmit cardiac MRI in pigs at 7T. J Magn Reson. 2019;305:195‐208. 31306985 10.1016/j.jmr.2019.07.004

[13] Schmitter S , DelaBarre L , Wu X , et al. Cardiac imaging at 7 tesla: single‐ and two‐spoke radiofrequency pulse design with 16‐channel parallel excitation. Magn Reson Med. 2013;70:1210‐1219. 24038314 10.1002/mrm.24935 PMC3960017

[14] Winter L , Kellman P , Renz W , et al. Comparison of three multichannel transmit/receive radiofrequency coil configurations for anatomic and functional cardiac MRI at 7.0T: implications for clinical imaging. Eur Radiol. 2012;22:2211‐2220. 22653280 10.1007/s00330-012-2487-1

[15] Terekhov M , Elabyad IA , Schreiber LM . Global optimization of default phases for parallel transmit coils for ultra‐high‐field cardiac MRI. PLoS One. 2021;16:e0255341. 34358243 10.1371/journal.pone.0255341 PMC8346258

[16] Scheffler K , Lehnhardt S . Principles and applications of balanced SSFP techniques. Eur Radiol. 2003;13:2409‐2418. 12928954 10.1007/s00330-003-1957-x

[17] Aigner CS , Dietrich S , Schaeffter T , Schmitter S . Calibration‐free pTx of the human heart at 7T via 3D universal pulses. Magn Reson Med. 2022;87:70‐84. 34399002 10.1002/mrm.28952

[18] Dietrich S , Aigner CS , Kolbitsch C , et al. 3D free‐breathing multichannel absolute B 1 + mapping in the human body at 7T. Magn Reson Med. 2020;85(5):2552‐2567. 33283915 10.1002/mrm.28602

[19] Chung S , Kim D , Breton E , Axel L . Rapid B1+ mapping using a preconditioning RF pulse with TurboFLASH readout. Magn Reson Med. 2010;64:439‐446. 20665788 10.1002/mrm.22423 PMC2929762

[20] Kent JL , Dragonu I , Valkovic L , Hess AT . Rapid 3D absolute B(1) (+) mapping using a sandwiched train presaturated TurboFLASH sequence at 7 T for the brain and heart. Magn Reson Med. 2023;89:964‐976. 36336893 10.1002/mrm.29497 PMC10099228

[21] Terekhov M , Elabyad IA , Resmer F , et al. Customized B1+‐Shaping using Multi‐Channel Transceiver Array Prototype for 7T Cardiac MRI with Central Elements Symmetry. 2020 Virtual Meeting.

[22] Terekhov M , Elabyad IA , Lohr D , Hofmann U , Schreiber LM . High‐resolution imaging of the excised porcine heart at a whole‐body 7 T MRI system using an 8Tx/16Rx pTx coil. MAGMA. 2023;36:279‐293. 37027119 10.1007/s10334-023-01077-z PMC10140105

[23] Terekhov M , Elabyad IA , Lohr D , et al. Complementary analysis of specific absorption rate safety for an 8Tx/16Rx array with central symmetry of elements for magnetic resonance imaging of the human heart and abdominopelvic organs at 7 T. NMR Biomed. 2023;36:e5023. 37620002 10.1002/nbm.5023

[24] Terekhov M , Aigner C , Dietrich S , Lohr D , Schmitter S , Schreiber LM . Tailored cost functions for improved static pTX‐based B1+ shimming in 7T cardiac MRI. 2023 Toronto, Canada.

