# Supplementary figures and images for: 7T 19F/ 1H MRI with perfluorocarbon‐labeled immune cells in pigs: Pilot results with a dedicated twin‐array system with pTX support
Source: Magn Reson Med. 2025 Oct 10;95(1):51–61. doi: 10.1002/mrm.70105 (PMC12620145; doi:10.1002/mrm.70105)

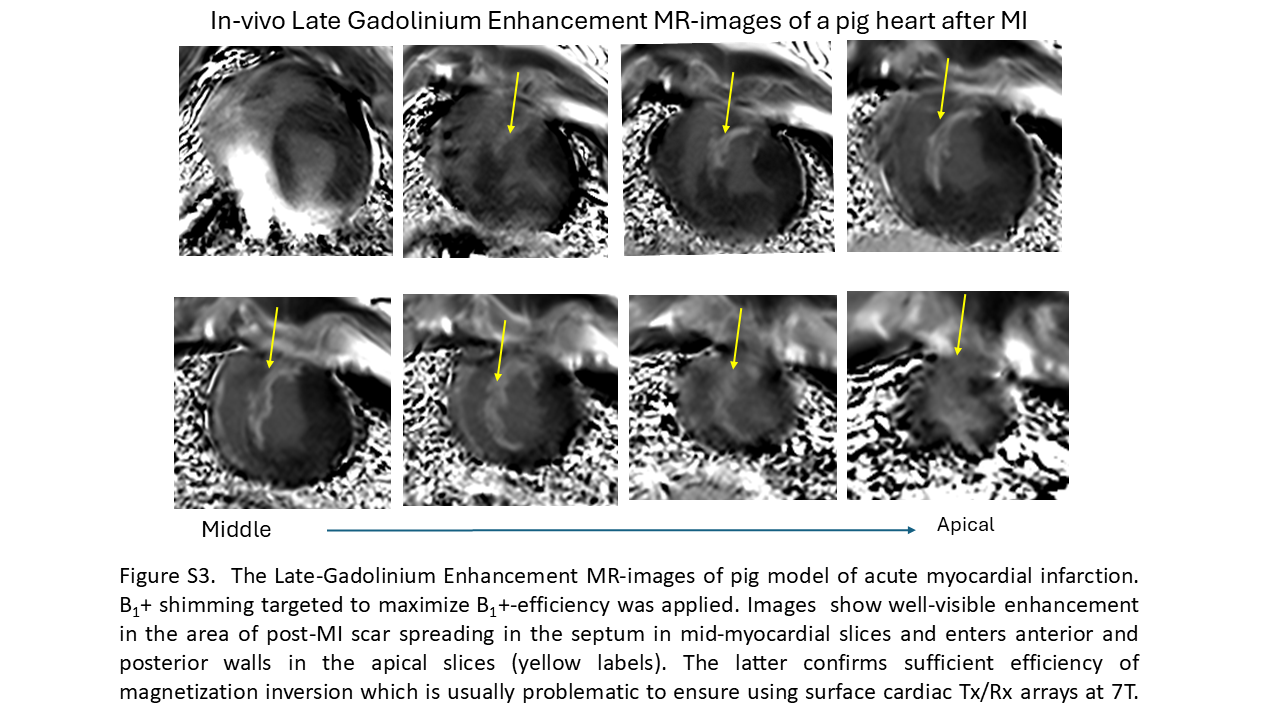

Supplement: Supplementary file 3 — Figure S1 The Late‐Gadolinium Enhancement MR‐images of a pig model of acute myocardial infarction. B1 + shimming targeted to maximize B1 +‐efficiency was applied. Images show well‐visible enhancement in the area of post‐MI scar spreading in the septum in mid‐myocardial slices and enters the anterior and posterior walls in the apical slices (yellow labels). The latter confirms sufficient efficiency of magnetization inversion, which is usually problematic to ensure using surface cardiac Tx/Rx arrays at 7T. [file MRM-95-51-s001.tif]
