# Supplementary material for: 7T 19F/ 1H MRI with perfluorocarbon‐labeled immune cells in pigs: Pilot results with a dedicated twin‐array system with pTX support
Source: Magn Reson Med. 2025 Oct 10;95(1):51–61. doi: 10.1002/mrm.70105 (PMC12620145; doi:10.1002/mrm.70105)
Supplement: Supplementary file 4 — Table S1. Pulse sequences and their parameters used for the experimental MR‐data acquisition. [file MRM-95-51-s003.docx]

| **Sequence** | **Resolution [mm]** | **FA [^0^]** | **TR [ms]** | **TE [ms]** | **TI [ms]** | **Application target** |
| --- | --- | --- | --- | --- | --- | --- |
| Rel. B_1_ GRE | 4x4x4 | 7 | 50 | 2.2 | N/A | B_1_^+^-mapping phantom |
| saturated turboFLASH | 4x4x4 | 7 | 12000 | 2.1 | N/A | B_1_^+^-mapping phantom |
| GRE ^1^H | 2x2x5 | 10 | 50 | 2.1 | N/A | Phantoms B_1_^+^-shimming test |
| GRE ^19^F | 5x5x8 | 15 | 400 | 2.1 | N/A | Phantoms B_1_^+^-shimming test |
| turboFLASH | 2.5x2.5x5 | 15 | 20 | 2.1 | N/A | g-factor mapping |
| BEAT CINE | 0.9x0.9x6 | 17 | Cardiac gated | 2.8 | N/A | Cardiac function CINE in-vivo, in-vivo B_1_^+^-shimming test |
| BEAT-IR | 0.9x0.9x6 | 17 | Cardiac gated | 2.8 | 300 | LGE invivo |
| GRE ^19^F | 5x5x8 | 15 | 400 | 2.1 | N/A | ^19^F MRI of nano-emulsion invivo and ex-vivo in the heart |
| DIR-GRE (MP2RAGE) | 0.65x0.65x0.65 | 7 | 3000 | 2.8 | 650/2700 | ^1^H MRI of the scar tissue in the explanted heart |

**Table 1** Summary of the pulse sequence parameters used in the presented work.
